# Effect of different delivery modes on the short-term strength of the pelvic floor muscle in Chinese primipara

**DOI:** 10.1186/s12884-018-1918-7

**Published:** 2018-07-03

**Authors:** Yun Zhao, Li Zou, Mei Xiao, Wan Tang, Hai-yi Niu, Fu-yuan Qiao

**Affiliations:** 1 Department of Obstetrics, Maternal and Child Health Hospital of Hubei Province, No 745 Wuluo Road, Wuhan City, People’s Republic of China; 2 Department of Obstetrics and Gynecology, Union Hospital of Tongji Medical College, Huazhong University of Science and Technology, Wuhan City, People’s Republic of China; 3 Department of Obstetrics and Gynecology, Tongji Hospital of Tongji Medical College, Huazhong University of Science and Technology, Wuhan City, People’s Republic of China

**Keywords:** Pelvic floor muscle strength, Cesarean delivery, Episiotomy, Perineal laceration, Forceps, Eletrical stimulation, Biofeedback

## Abstract

**Background:**

To investigate the effect of different delivery modes and related obstetric factors on the short-term strength of the pelvic floor muscle after delivery in Chinese primipara.

**Methods:**

A total of 4769 healthy Chinese primiparas at postpartum 6–8 weeks were interviewed. According to the difference of delivery mode, the selected primiparas were divided into 2 groups, including cesarean delivery group containing 2020 and vaginal delivery group containing 2749. All the vaginal deliveries were further divided into 3 groups, including episiotomy group containing 2279, perineal laceration group containing 398, and forceps assisted group containing72. The scales of their pelvic floor muscle (PFM) strengths were examined by specially trained personnel using digital palpation (Modified Oxford scale:0–5 grade). According to participants’ willingness, if the PFM strength was weak (0 or 1 grade), at-home PFM training would be recommended and an electrical stimulation combined with biofeedback therapy would be conducted for them in hospital. Twelve weeks after delivery, the PFM strength would be measured again. For statistical analysis, *t*-test, one-way variance analysis, Chi-square analysis, Kruskal-Wallis test H, Mann-Whitney U test and Wilcoxon test were carried out.

**Results:**

The PFM strength in cesarean delivery group was higher than in vaginal delivery group (*p* < 0.05). Among 3 vaginal delivery groups, the PFM strength in perineal laceration group was the highest (*p* < 0.05); however, there was no difference in PFM strength between episiotomy group and forceps assisted group (*p*>0.05). After accepting PFM training at home and therapy in hospital, 305 women showed increased PFM strength (*p* < 0.05).

**Conclusions:**

Vaginal delivery is an independent risk factor causing the damage of PFM, and episiotomy may cause injury of PFM. Through PFM training at home and therapy in hospital, those damage will resume as soon as possible in the short-time period after delivery.

**Electronic supplementary material:**

The online version of this article (10.1186/s12884-018-1918-7) contains supplementary material, which is available to authorized users.

## Background

Pelvic floor disorders (PFDs) are common and prevalent in adult women, which negatively affect women’s self-perception of their body image and life quality [1] and bring great economic burden to patients and society. PFDs has become a public health problem attracting worldwide attention. According to recent epidemiological studies, pregnancy and childbirth are two independent factors causing PFDs [2]. Can cesarean delivery really protect pelvic floor function of women? Some studies have shown that the selective cesarean delivery may have a protective effect for the pelvic floor, but others have shown that the protective effect is very limited in the long term postpartum period [3]. How do different delivery modes affect female Pelvic Floor Muscle(PFM) and what are the effects of these delivery modes on Chinese primiparas in the short-term postpartum? This study was conducted to estimate the effect of different delivery modes on the PFM strength at postpartum 6–8 weeks and the refection of PFM training on the weak PFM strength in the short-time period after delivery. The scale of PFM strength was examined by specially trained personnel using digital palpation.

## Methods

### Ethical approval

The study protocol was approved by the Ethics Committee of Maternal and Child Health Hospital of Hubei Province(201301) and all included women signed written informed consent.

### Selection of patients and study design

**Fig. 1:**
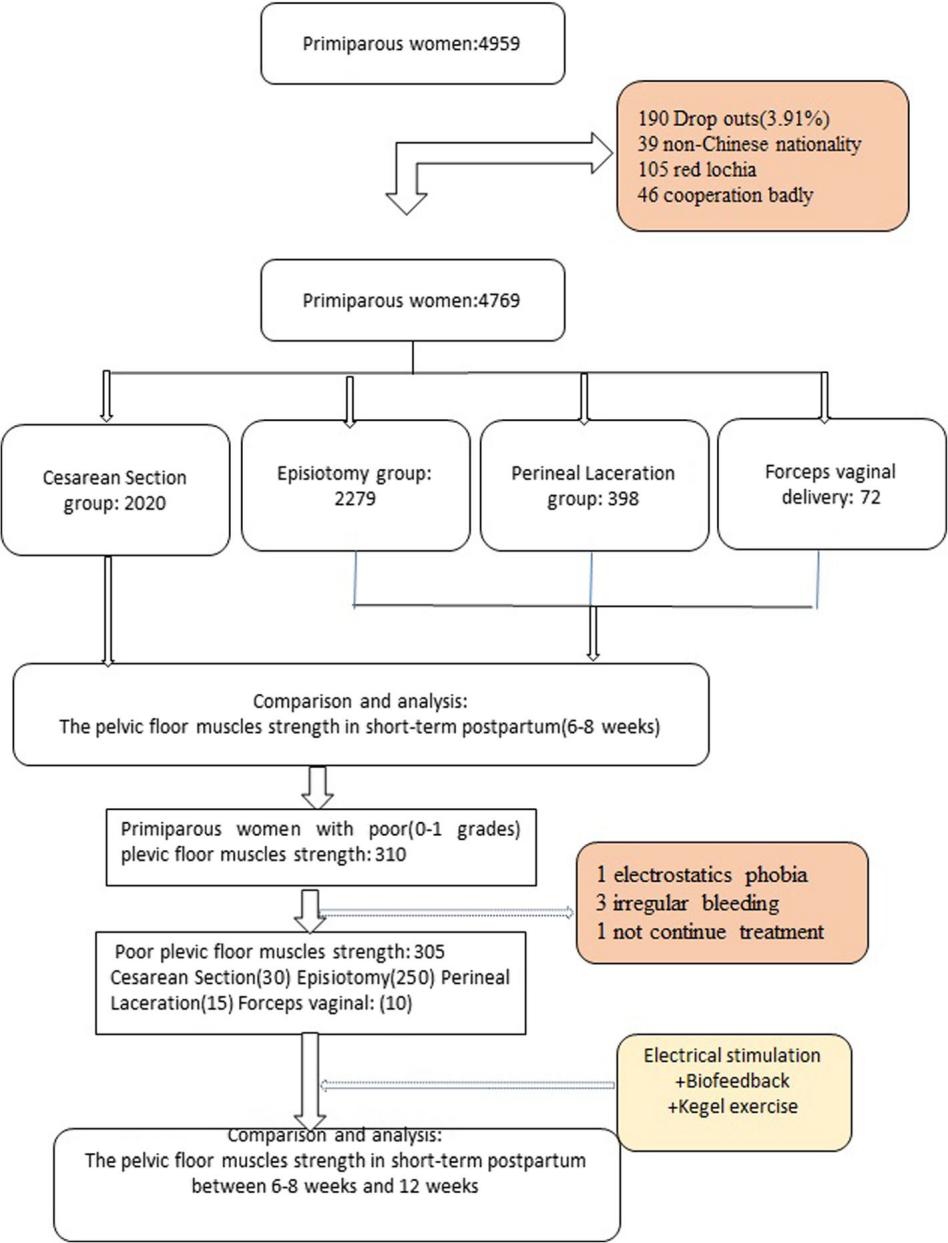
Flowchart demonstrating. The number of Participants who attended and the reasons for lost to follow-up

Women who had delivered in the obstetric department at the Maternal and Child Health Hospital of Hubei Province from January 2013 to January 2014 and visited pelvic floor rehabilitation department in postpartum 6–8 weeks period were selected as research objects. All selected women were healthy Chinese primiparas, with age ranging from 20 to 35 years old. They had clean lochia without obstetric complications. Exclusion criteria included those with age less than 20 years old or more than 35 years old, non-Chinese nationality, multiple pregnancies, vaginitis and urinary tract infection, and mental incapacity. A total of 4959 women were selected in this study, excluding 39 for being non-Chinese nationality, 105 for having red lochia, 46 for not cooperative with specially trained personnel. According to delivery mode, 4769 cases were divided into 2 groups, including cesarean delivery group containing 2020 and vaginal delivery group containing 2749. Then, All vaginal deliveries were further divided into 3 groups, including episiotomy group containing 2279 (Left lateral episiotomy during vaginal delivery), perineal laceration group containing 398 (I, II degree natural perineal laceration during vaginal delivery), and forceps assisted group containing 72 (Left lateral episiotomy and forceps assisted operation during vaginal delivery). In our hospital, all instrument–assisted operations of vaginal delivery were carried out with the aid of forceps in place of vacuum-assisted delivery, and midwifery care was conducted during the antenatal period, labor and delivery period, and postnatal period according to The National Midwifery Guidelines. The evaluation of pelvic floor muscle strength and routine physical examination on maternal gynecological situation were performed by trained specialists. All the investigation and examination results were recorded truthfully, including patient characteristics, medical history, and pregnancy delivery data. All selected women had not accepted formal training of pelvic floor muscle during pregnancy and postpartum 6–8 weeks period (see Fig. 1).

According to participants’ actual condition in the 6–8 postpartum weeks, if the PFM strength was weak (0 or 1 grade according to modified Oxford scale), at-home PFM training plus in-hospital electrical stimulation combined with biofeedback therapy would be recommended for them, that was guided by specialized medical personnel in our department. Twelve weeks after delivery, the PFM strength would be examined again. There were only 310 primiparas with weak PFM strength who were treated with at-home PFM training plus in-hospital electrical stimulation combined with biofeedback therapy. Among them, excluding 1 case having electrostatic proble phobia when accepting electrical stimulation for the first time, 3 cases showing irregular vaginal bleeding, 1 case failing to continue treatment, the remaining 305 cases were included in the study for observing the therapeutic effect of PFM training at home plus PFM therapy in hospital.

### Determination of pelvic floor muscle strength

Pelvic floor muscle was determined by specialized medical personnel using digital palpation. Before the examination, women were told to empty the bladder and take lithotomy position. PFM contraction without any movement of the pelvis or visible contraction of the glutei, hip, or abdominal muscles was emphasized [4].

Digital palpation [5]. First put the index and middle fingers 2–3 cm deep into the vagina and identify the levator ani muscle. Then, separate the two fingers and fall on two sides of levator ani muscle, meanwhile, put the other hand on the abdomen to make sure the abdominal muscle was relaxed. The scale of pelvic floor muscle strength was described by Modified Oxford scale. On the basis of contraction strength and retraction capability, the PFM strength was divided into 0–5 grades and 6 classes. (Table 1).

**Table 1:** Modified Oxford scale for digital evaluation of pelvic floor muscle strength

Grade	Description
0	Nil
1	Flicker
2	Weak
3	Moderate, slight lift of the examiner’s fingers, no resistance
4	Good, sufficient to elevate the examiner’s fingers against light resistance
5	Strong, sufficient to elevate the examiner’s fingers’ against strong resistance

### PFM training [6]

When the scale of PFM during 6–8 weeks postpartum period was < 2 grades (0 or 1 grade), the specialists in our department of pelvic floor rehabilitation would teach them how to contract their PFM by demonstrating vaginal palpation. At first, the specialist placed her index finger into the vaginal and told the women to lift and squeeze around the finger. Then, the women took Kegel exercise 10 times a day at home, each exercise included 10 repetitions. After 12 weeks of postpartum, the woman was told to come back again for PFM strength examination.

### Eletrical stimulation and biofeedback [7]

The PFM enforcement program consisting of biofeedback and electrical stimulation therapy, lasting 30 min, was implemented twice a week. A complete course of PFM enforcement program lasts for 5 weeks, which is 10 times as long as the program of biofeedback and electrical stimulation. MyoTrac infiniti (SA9800) device (Thought Technology Inc., Montreal, Canada) was adopted in this experiment. The biostimulation feedback system is based on biofeedback technology, bioformatics principles and bioengineering technology, and so on. Safe and effective electrical stimulation combined with biofeedback technology were conducted by vaginal sensor to record pelvic floor muscle activity and pelvic floor muscle contraction strength, based on which the strength of electrostimulation was controlled. The direct vagina low-voltage low-frequency electric stimulation including 3 reference electrodes on the iliac and two sides hypogastric skin was performed to prevent the abdominal or giuteus musles contraction. A separate proble was used for primiparas to prevent cross-infection. With the help of computer graphics, various parameters of the device was adjusted to make every primipara feel the PFM active and passive contraction and guarantee the primiparas not feel any pain or discomfort. The parameters of electrical stimulation were set as pulse width of 20–40 us and low voltage of 40-80 Hz. The electrostimulation included rapid contraction exercise of PFM (stimulation and interval time is 2 s:2 s) and continuous contraction exercise of PFM ((stimulation and interval time is 5–8 s:10 s). The electrical stimulation and biostimulation feedback therapies were applied alternatively based on the needs of primiparas with weak PFM strength. Each treatment of electrical stimulation combined with biostimulation lasted for 30 min, and was conducted two times per week, 10 times for a complete course of treatment.

### Statistical analyses

All the statistical analyses were carried out using the Statistical Package of Social Sciences software (SPSS Version 13.0 Inc., Chicago, IL, USA). The values and variables were reported in the form of mean ± standard deviation. One-Way ANOVA and Student’s test were performed to compare the variables in Gaussian distribution. Chi-square test was used to evaluate the categorical variables. Kruskal-Wallis H test was used to assess the difference of the PFM function and the variables in no Gaussian distribution among K independent samples. Mann-Whitney U test and Bonferroni’s correction were conducted to assess further pairwise samples. Wilcoxon test was used to evaluate the difference of the PFM function before and after PFM training. The difference was considered statistically significant at *p* < 0.05.

## Results

### Demographic data of cesarean delivery group and vaginal delivery group

Through statistical comparison, it can be known that the difference is not statistically significant between cesarean delivery group and vaginal delivery group (*p* > 0.05). Samples in cesarean delivery group have older age, heavier new baby’s weight, and higher rate of gestational diabetes mellitus(GDM) than that in vaginal delivery group (*p* < 0.05). The demographic data of two groups are list in Table 2.

**Table 2:** Comparison of demographic data among Cesarean delivery and three groups of vaginal delivery

	Cesarean delivery Group	Vaginal delivery Group	t or X^2^	*p* value	Perineal laceration Group	Episiotomy Group	Forceps assisted Group	F or X^2^	*p* value
	(2020)	(2749)			(398)	(2279)	(72)		
Age(y) [mean ± sd]	28.3 ± 3.0	27.7 ± 2.9	7.017	0.000	27.7 ± 2.8	27.7 ± 2.9	28.1 ± 2.8	0.639	0.528
BMI of delivery (kg/m^2^) [mean ± sd]	27.3 ± 2.6	27.5 ± 2.7	−1.658	0.098	27.5 ± 2.8	27.4 ± 2.7	27. 8 ± 2.6	0.656	0.519
Birth weight(g) [mean ± sd]	3322.0 ± 550.0	3252.6 ± 476.1	4.554	0.000	3226.3 ± 334.2	3254.8 ± 499.1	3329.2 ± 385.0	1.563	0.210
Gestational weight gain(kg)[mean ± sd]	15.9 ± 7.0	15.6 ± 7.2	1.470	0.142	15.6 ± 6.7	15.6 ± 7.3	14.6 ± 6.2	0.639	0.528
Gestational age at birth(w) [mean ± sd]	39.2 ± 1.4	39.3 ± 1.4	−0.638	0.523	39.2 ± 1.3	39.3 ± 1.4	39.7 ± 1.1	2.135	0.094
Rate of GDM (%)	30.0 (605/2020)	26.0 (714/2749)	9.009	0.003	24.1 (96/398)	26.0 (593/2279)	25 (18/72)	0.660	0.719
Duration of 2nd sta(m) [median(95%CI)]	/	26(7–110)	/	/	29(6–77)	25(7112)	28(11–129)	4.258	0.119

Student’s test, One-Way ANOVA, Kruskal-Wallis H test and Chi-square analysis are performed; GDM: gestational diabetes mellitus

### The PFM strength of women in postpartum 6–8 weeks after accepting cesarean delivery or vaginal delivery

Table 3 shows that the PFM strength of women in cesarean delivery group is stronger than in vaginal delivery group, and the difference is statistically significant (*p* < 0.05).

**Table 3:** The PFM strength of women in 6–8 weeks postpartum betwee the two delivery modes

	N	0	1	2	3	4	5
Cesarean secarean Group	2020	38	232	1364	366	20	0
Vaginal delivery Group	2749	223	1184	1195	140	7	0
Z	−27.861						
*p* value	0.000						

Mann-whitney U test is performed

### Demographic data of the three groups of vaginal delivery

Through statistical comparison, it can be known that there are no differences in age, BMI of delivery, new baby’s birth weight, gestational weight gain, gestational age at birth and the rate of GDM among episiotomy group, perineal laceration group, and forceps assisted group (*p* > 0.05). The median duration of the 2nd stage in forceps assisted group was 28 m(95%CI, 11-129 m), there was no statistical significance among the three groups (*p* > 0.05).The rates of 0, 1 and 2 degree of perineal lesions in perineal laceration group was 1.3, 26.4%(105/398) and 72.4%(288/398). The rates of 2 degree of perineal lesions in episiotomy group and in forceps assisted group were 100%, there was no 3 or 4 degree of perineal lesions in any of the three groups of vaginal delivery. There was common preineum local block anesthesia before episiotomy but no neuraxial labor anlagesia for vaginal delivery. The demographic data of 3 vaginal delivery groups are list in Table 2.

### The PFM strength of women in postpartum 6–8 weeks among three groups of vaginal delivery

Table 4 shows that the PFM strength of women among three vaginal delivery groups is significant different (*p* < 0.05). The PFM strength in perineal laceration group is stronger than that in episiotomy group and forceps vaginal delivery group, and the intergroup differences are statistically significant (*p* < 0.025). There is no significant difference in PFM strength of women between episiotomy group and forceps vaginal delivery group (*p*>0.05).

**Table 4:** The PFM strength of women in 6–8 weeks postpartum among the different vaginal delivery modes

	N	0	1	2	3	4	5
Perineal laceration Group①	398	21	151	192	30	4	0
Episiotomy Group②	2279	197	996	975	108	3	0
Forceps assisted Group③	72	5	37	28	2	0	0
	①②③		①-②	①-③		②-③	
X^2^ or Z	18.736		−4.182	−2.550		−0.830	
*p-*value	0.000		0.000	0.032		1.000	

Kruskal-Wallis H test was used to assess the difference of the PFM function among the three vaginal delivery modes

Mann-Whitney U test and Bonferroni’s correction were conducted to assess pairwise samples

### The weak PFM strength of women in postpartum 6–8 weeks

There were 305 cases with weak PFM willing to receive at-home PFM exercise plus electrical stimulation combined with biofeedback therapy in hospital twice a week (10 times for a complete course). Among them, there were 30 cases of cesarean delivery, 250 cases of were episiotomy, 15 cases of perineal laceration, and 10 cases of forceps vaginal delivery. By the last time of their treatment (nearly 12 weeks postpartum), their PFM strengths were examined again, and it could be found that their PFM strengths were improved (*p* < 0.05), as shown in Table 5.

**Table 5:** Comparison with the PFM strength before and after treatment

	n	0	1	2	3	4	5
Before	305	208	97	0	0	0	0
After	305	0	51	202	40	12	0
Z		−15.572					
*p*-value		0.000					

Wilcoxon test is used

## Discussion

Pregnancy and childbirth are two important events in a women’s life. The two phases of life have been proved to be associated with increased incidence of PFDs. Pregnancy and childbirth, especially vaginal childbirth, are two independent factors causing PFDs [8, 9]. Although severe morbidity of women from PFDs is rare, PFDs do seriously affect the quality of women’s life. At present China, permanent damage of PFM in older women has attracted much attention and lots of money have been invested on this issue. However, the prevention and treatment of PFDs during pregnancy and childbirth have not been attached with sufficient attention. The postpartum period is the time during which the PFM damage of women may develop into the most serious condition (fortunately it is reversible). If a woman in postpartum cannot persist on accepting conservation treatment (pelvic muscle exercise, electrical stimulation, and biofeedback, and so on) for postnatal PFDs, permanent PFDs will gradually come into being [10, 11]. Paying attention to PFM damage and PFDs in recent postpartum period as well as finding effective method to guide restoration of pelvic floor function and improving the quality of women’s life are the direction of our efforts in the future.

At present, common detection methods of PFM strength include digital palpation, vaginal balloon, and surface electromyography (SEMG) and so on. Digital palpation can quantify the PFM strength directly, although it has always been questioned for its subjectivity. Vaginal balloon is more objective but not as precise as digital palpation. SEMG detects the electrical activity of PFM, yet its reliability in clinical application is still controversial. There is a strong correlation among the three methods in the assessment of the PFM strength [12–14]. Digital palpation is mainly used in the census, while SEMG is normally used in evaluation of therapeutic effect of the PFM strength [14]. In this study, through census of the PFM strength of women in postpartum 6–8 weeks, it can be known that the PFM strength of women having cesarean delivery is higher than that of women having vaginal delivery (including perineal laceration, episiotomy, and forceps assisted vaginal delivery), although t primiparas having cesarean delivery have older age, heavier baby’s weight and higher rate of GDM than those having vaginal delivery. This shows vaginal delivery is one of the important factors causing PFM damage in recent postpartum, which is consistent with results of previous researches [15, 16]. This study shows that the PFM strength of primiparas having perineal laceration vaginal delivery is higher than that of primiparas having episiotomy vaginal delivery or forceps assisted vaginal delivery, which is mainly due to that the episiotomy may damage PFM and the integrity of pudendal nerve. It has always been a controversial topic to perform routine episiotomy on low-risk pregnant women during vaginal delivery [17–19]. Supporters think that routine episiotomy can protect the anal sphincter [18]. Opponents argue that routine episiotomy can increase the rates of postpartum bleeding, postpartum perineal incision infection, postpartum pain, urinary morbidity, and so on [19]. Our study suggests that routine episiotomy cannot protect the PFM from being damaged. The PFM damage is also related with PFDs, therefore the effective measure to reduce and prevent PFDs on low-risk pregnant women is to protect the perineal integrity and do not perform routine episiotomy during vaginal delivery. Many studies show that operation vaginal delivery by forceps or vacuums significantly damage the PFM strength and increase the risk of PFDs during both the recent and long term postpartum period [20–22]. But our study shows that there is no significant difference between episiotomy and forceps assisted vaginal delivery. This may be due to that the rate of forceps assisted vaginal delivery is very low in China, which is 1.51% (72/4769) in our hospital of primiparous women; for comparison, the percentage of operative vaginal delivery is 29.1% among primigrous women in Ireland [23]. The fetal head position will be lower or outlet when using forceps, and some difficult forceps vaginal deliveries have been replaced by cesarean delivery in our hospital. Vaginal delivery is an independent risk factor leading to PFDs of primiparous women in short-time postpartum period. Although cesarean delivery reduces the risk of pelvic floor trauma, it is not entirely protective [24]. Cesarean delivery is a protective delivery method for PFM of women in recent postpartum, but may be not useful for women in late postpartum [17]. Moreover, cesarean delivery may easily bring other severe complications. Episiotomy may cause injury of PFM in low risk vaginal delivery or forceps vaginal delivery. We should try our best to reduce the rate of episiotomy in order to protect PFM in primiparous women.

When the damage of PFM occurs, how to restore and exercise the weak PFM in the short-time postpartum period is the focus. Many studies show that the PFM training can prevent and treat pelvic floor disease in antenatal and postnatal women [25]. Our study shows that the combined treatment of at-home PFM exercise plus in-hospital electrical stimulation combined with biofeedback is an effective way to restore the PFM strength that is damaged by pregnancy and delivery in a short-time.

### The limitation of our study

Our study is subjected to a few limitations. Firstly, the modified Oxford score is very simple and is not sensitive enough to describe accurate change of PFM strength of the postpartum women. Another limitation is that palpation does not provide global assessment of the levator ani muscle which can be measured by techniques such as ultrasound imaging, MRI, a predefined protocol [26], and so on. Lastly, due to lack of experience, it is difficult for postpartum women to properly cooperate with examination expert.

The high rate of cesarean delivery and low rate of operative vaginal delivery are the limitations of our study. In China, the high cesarean delivery rate is a social problem, nearly half of all newborns in China are delivered by cesarean approach [27]. From January 2013 to January 2014, there were 13,490 cases of Chinese s primiparas delivered in our birth centre, the overall rate of cesarean delivery was 43.1%(5809/13490) and the overall rate of operative vaginal delivery was 1.6%(220/13490). Many operative vaginal deliveries were replaced by cesarean delivery, and the rate of maternal request cesarean delivery was nearly 10.0%(1351/13490). There was common preineum local block anesthesia before episiotomy but no neuraxial labor anlagesia for vaginal delivery.

## Conclusions

Vaginal delivery is an independent risk factor causing the damage of PFM in the short-term after delivery. Episiotomy may cause injury of PFM. Through PFM training at home plus electrical stimulation combined with biofeedback therapy in hospital, those damage will resume as soon as possible in the short-time period after postpartum.

## Additional file

Additional file 1: Individual patient data. (XLSX 304 kb)

## Abbreviations

GDM: Gestational diabetes mellitus
PFDs: Pelvic floor disorders
PFM: Pelvic floor muscle
SEMG: Surface electromyography
